# Development of Immunoassays Using Interferometric Real-Time Registration of Their Kinetics

**Published:** 2014

**Authors:** A. V. Orlov, A. G. Burenin, V. O. Shipunova, A. A. Lizunova, B. G. Gorshkov, P. I. Nikitin

**Affiliations:** Prokhorov General Physics Institute, Russian Academy of Sciences, Vavilov Str., 38, 119991, Moscow, Russia; Moscow Institute of Physics and Technology, Institutskiy per., 9, 141700, Moscow Region, Dolgoprudny, Russia

**Keywords:** Label-free biosensors, interferometry, sensor chips, surface functionalization, surface epoxylation, surface biotinylation, efficiency of biomolecular immobilization, immunoassay, magnetic nanoparticles, cardiac troponin I

## Abstract

A method for effective development of solid-phase immunoassays on a glass surface and for optimization of related protocols by highly sensitive quantitative monitoring of each assay step has been proposed and experimentally implemented. The method is based on the spectral correlation interferometry (SCI) that allows real-time measuring of the thickness of a biomolecular layer bound to the recognition molecular receptors on the sensor chip surface. The method is realized with compact 3-channel SCI-biosensors that employ as the sensor chips standard cover glass slips without deposition of any additional films. Different schemes for antibody immobilization on a glass surface have been experimentally compared and optimized toward a higher sorption capacity of the sensor chips. Comparative characterization of the kinetics of each immunoassay stage has been implemented with the optimized protocols: i) covalent immobilization of antibody on an epoxylated surface and ii) biotinylated antibody sorption on a biotinylated surface via a high-affinity biotin-streptavidin bond. We have shown that magnetic nanoparticles employed as labels with model detection of cardiac troponin I further amplify the SCI signal, resulting in 100-fold improvement of the detection limit. The developed protocols can also be used with the alternative immunoassay platforms, including the label methods based on registration of only the final assay result, which is the quantity of bound labels.

## INTRODUCTION

In recent years much attention has been focused on studies of methods for the identification of the protein markers of diseases in complex biological fluids such as blood, serum, saliva and others. The development of immunoassay methods for detection of these substances is of high importance for clinical [1] and emergency [2] diagnostics, control of treatment efficiency [3], discovery of new specific antigens as disease markers [4], drug development [5-7], etc. One of the most common immunoassay formats [8] that provides high sensitivity, accuracy, and specificity is the solid phase sandwich immunoassay [9, 10]. It is based on formation of a “capture antibody-antigen-tracer antibody” complex on the solid phase, which is possible only in the presence of the antigen under test. The result is recorded with the use of different labels (enzymatic [11], latex [12], gold [13], magnetic [14, 15] and others) conjugated with tracer antibodies either covalently or by a highly affine intermediate bond, e.g., antibody-antigen [16], biotin-streptavidin [17], or barnase-barstar [18, 19].

The key characteristics of immunoassays (detection limit, linear and dynamic range, sensitivity, and specificity) depend on the selected antibodies, mode of their immobilization on the solid phase, square of the solid phase surface, incubation time and concentration of immunoreagents, as well as on the composition of the buffer and stabilizing solutions [20]. If one uses the label-based methods, the contribution of each of the mentioned parameters can be estimated only at the final stage of the immunoassay. Label-free optical methods can substantially increase the efficiency of development of immunoassay protocols due to real-time monitoring of all stages of the biochemical reactions and through reducing the assay duration and number of operations. The methods that employ expensive sensor chips with precisely deposited gold films [21, 22], optical dielectric films with a regulated refractive index [23], porous silicon structures with a fixed porosity depth [24] can be named among the most commonly used techniques. As a result, many methods are too costly compared to the conventional ELISA for diverse applications that require disposable consumables.

Earlier, we proposed the original methods of spectral phase [25, 26] and spectral correlation interferometry [27-29] for registration of biomolecular interactions on the surface of plane-parallel transparent plates, e.g., inexpensive glass cover slips, either without any coating or coated with thin films typical for the surface layers of biosensor chips. These methods were successfully employed for quantitative detection of conformational changes in polymers [30], identification of disease markers in blood serum [31], pyrethroids in environmental monitoring [32], and discovering the functional mechanisms of drugs [33].

This work was aimed at developing a method for optimization of sandwich-type immunoassays using realtime monitoring of each assay stage with the spectral correlation interferometry. Optimization of the magnetic immunoassay [15, 34, 35] that employs magnetic nanoparticles as labels has been performed as an experimental demonstration of the developed method.

## EXPERIMENTAL

**Reagents**

A complex of subunits of the cardiac troponins I, T and C, monoclonal antibodies to cardiac troponin I (clones 19C7 and 16A11), conjugates of monoclonal antibodies to cardiac troponin I (clones 19C7 and 16A11) with biotin were kindly provided by prof. A.G. Katrukha (Immunology Group at Moscow State University, Moscow). (3-Aminopropyl)triethoxysilane, (3-glycidyloxypropyl) trimethoxysilane, and biotin N-hydroxysuccinimide ester were purchased from Sigma Aldrich (USA); commercially available nanoparticles of ~50 nm composed of several crystals of ferric oxide and covered with a polymeric coating with covalently conjugated recognition biomolecules of streptavidin or monoclonal rat anti-mouse antibodies to isotype IgG1 were obtained from Miltenyi Biotec (Germany) . Other reagents were at least of analytical grade.

**Characterization of magnetic nanoparticles (MNP)**

Microphotographs of MNP were made using a JEOL JEM-2100 transmission electron microscope with an accelerating voltage of 200 kV.

**The method of spectral correlation interferometry (SCI)**

The spectral correlation interferometry (SCI) method described in detail in Refs. [27-29] employs two Fabry– Perot interferometers in an original optical setup with the use of superluminescent diode radiation. The first interferometer base (distance between the mirrors) is periodically changed by a piezoelectric driver. A transparent plane-parallel plate made, for instance, of glass or plastic without any coating or with partially transparent films deposited on its surface, serves as the second interferometer and, simultaneously, as the sensor chip. In this work, a glass cover slip with immobilized receptor molecules on its surface is used as the sensor chip. It has been shown that such slips with a thickness of 100 µm can perform as acceptable Fabry–Perot interferometers if the size of each separate registration area is 2–8 mm. Within such a distance, the thickness variations of the standard cover slips are less than a quarter of the radiation wavelength.

**Fig. 1:**
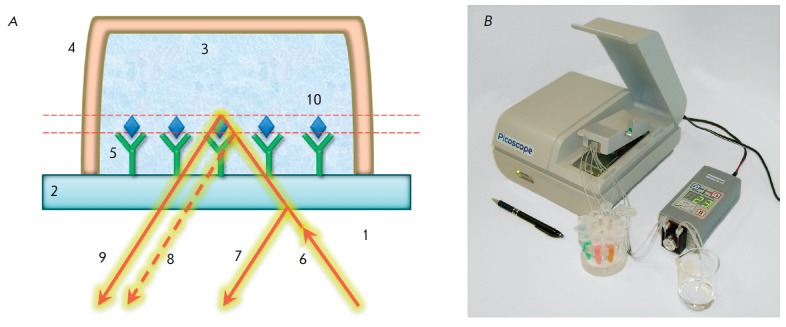
The SCI principle. Changes in the optical thickness of a biolayer on a glass surface are recorded by a spectrum of interfering beams reflected from the sensor chip (A): 1 – air; 2 – microscopic glass cover slip; 3 – test solution; 4 – flow channel; 5 – receptor molecules; 6 – incident beam of superluminescent diode; 7,9 – reflected beams; 8 – position of the reflected beam before a biochemical reaction; 10 – detected biomolecules. (B): Photo of a three-channel Picoscope^®^ biosensor

The SCI method uses the interference between a reference beam reflected from the lower surface of the cover slip and a probe beam reflected from the upper glass surface with biorecognition molecules (*Fig. 1A*). During the biochemical reaction under study, biomolecules from the solution (ligands) bind to the receptor molecules on the sensor chip, thus increasing the optical path of the probe beam reflected from the “liquidbiolayer” interface. The result of interference of these two beams depends on the biological layer thickness, whose variations during the reaction are calculated using the changes in the phase of the correlation signal while scanning the base of the first interferometer.

The SCI method is realized as the Picoscope^®^ family of devices (*Fig. 1B*), which allows real-time registration of the dynamics of the molecular reactions on the surface of the cover slips with the picometer resolution of thickness averaged over the observation spot [28]. In the used instrument realization, the sensor chip was placed inside the device and covered with a cuvette that provided three independent channels. Each channel was connected to a flow system that provided the reagent supply along the upper surface of the slip. The channel height was 0.1 mm; linear dimensions were 3.5 × 1.7 mm. The reagents were supplied at 7.5 µl/min at room temperature. The optical detection in each channel was performed using the spectrum of interfering beams reflected from the lower surface of the sensor chip in a registration spot of 1 mm^2^ in the central part of the channel. Picoscope® allows one to use the sensor chips with preliminarily immobilized antibodies [28]. In this work, the immobilization was performed directly in the flow inside the device to permit quantitative monitoring.

**Cleaning the glass slip surface**

The chemical modifications of the glass surface described below were performed according to the techniques developed for the detection of the cardiac troponin concentration based on the approaches discussed in [32].

In order to clean and increase the density of hydroxyl groups on the surface, the cover slips were washed with methanol and immersed in a 1 : 3 solution of 30% hydrogen peroxide and 95% sulfuric acid for incubation for 40 min at 70°C. The slips were then washed thrice with tri-distilled water and twice with methanol. After the cleaning, the slips were immediately subjected to further chemical modification.

**Amination of the glass slips and antibody immobilization**

Aminated glass slips were prepared as follows: the cleaned glass slips were immersed in a 3% APTE S solution in methanol and incubated overnight at room temperature, washed thrice in isopropanol, and dried. The aminated slips were stored at room temperature until usage.

For covalent immobilization, 5 µl of the antibody (1 mg/ml) was mixed with 1 mg of 1-ethyl-3-(3- dimethylaminopropyl)carbodiimide (EDC), 2 mg Nhydroxysuccinimide (NHS) and 35 µl of a 10 mM phosphate buffer (pH 5.0), and incubated for 15 min. Next, 160 µl of phosphate buffered saline (PBS), pH 7.4, was added and the resulting solution was passed over the surface of the aminated glass slips in the flow system of the Picoscope^®^ biosensor for 10 min.

**Epoxylation of the glass slips and antibody immobilization**

The clean glass slips were immersed in a 5% GLYMO solution in methanol and incubated for 16 h at room temperature. The slips were then washed thrice in isopropanol and dried in an exsiccator for 1 h at 105°C. The slips were stored at room temperature until usage. Immobilization of antibodies was performed directly in the flow system of the device by passing the respective antibody solution of 25 µg/ml in PBS over the glass slip surface.

**Biotinylation of the glass slips and antibody immobilization**

The aminated glass slips were immersed in a solution containing a 10 mM biotin N-hydroxysuccinimide ester and 500 mM triethylamine in dimethylformamide (DMF) for 2 h at room temperature. Following washing with DMF and methanol, the slips were dried and stored at room temperature until usage. For antibody immobilization, the solutions of streptavidin and the respective biotinylated antibody both at a concentration of 25 µg/ml in PBS were consecutively passed over the glass slip surface in the flow system of the device, both processes being monitored by a sensogram that showed the thickness increase of the biomolecules on the surface.

**Carboxylation of the glass slips and antibody immobilization**

The aminated glass slips were immersed in a solution containing 15 mM succinic anhydride in DMF for 2 h. After triple washing in DMF, the slips were dried and stored at room temperature until usage. Then, the slips were immersed in a mix of 10 mM EDC and 15 mM NHS in DMF for 15 min. After washing in DMF, the slips were dried. The sorption of the capture antibody at a concentration of 25 µg/ml in PBS was performed in the flow system of the device, along with control of the layer thickness averaged over the sensing area.

**Immunoassay for troponin detection**

The following immunoreagent solutions in PBS (pH 7.4) were consecutively passed over the biotinylated surface of the sensor chip: 1) streptavidin – 25 µg/ml, 2) biotinylated capture antibody (clone 19C7) to troponin – 25 µg/ml, 3) a complex of subunits of the cardiac troponins I, T and C with the addition of 100 µg/ml BSA and 0.01% glycine, 4) tracer antibody (clone 16A11) to troponin – 25 µg/ml, and 5) magnetic particles with 0.1% BSA. Between the immunoreagents, PBS was passed for 3 min for washing. The immunoassay on the aminated, epoxylated, and carboxylated surfaces of the sensor chips was done in the same way but a 25 µg/ml solution of the native capture antibody (clone 19C7) to troponin was passed instead of steps 1 and 2 . The detection limit was calculated by the 2σ criterion as the minimal antigen concentration at which the recorded signal exceeded the signal of the negative control in the absence of the antigen for at least two values of the standard deviation of the signal of the negative control.

**Determination of the observed kinetic association constant**

Determination of the observed kinetic association constant was based on the theoretical model of equilibrium association [32] adapted to the biosensor system in use. The values of the kinetic constants of association* k*_a_ and dissociation *k*_d_ as well as of the maximal signal* R*_max_ were chosen to provide best fitting of the observed sensogram sections during passing the analyte at concentration* C *to the approximating function:

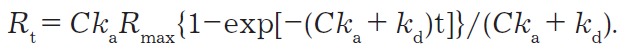



## RESULTS AND DISCUSSION

**Subject of the study**

Cardiac troponin I (cTnI) was chosen as a model antigen for demonstration of the immunoassay. cTnI is a specific marker of myocardial infarction [37]. It is localized in the cardiac muscle and participates in the regulation of its contraction. Upon injury of the cardiac muscle, troponin I enters the bloodstream [38], and its presence in blood allows one to distinguish acute myocardial infarction from other diseases with similar symptoms. The normal presence of this cardiomarker in the blood of healthy donors estimated by the 99th percentile of the control group slightly varies between tests from different manufacturers and accounts for 0.01–0.1 ng/ml [39]. Troponin concentration starts increasing in the first hours from myocardial infarction, reaches its peak in 24–48 h, when it can exceed 1000 ng/ml [40], but then returns to a normal level after 5–14 days [41]. Moreover, this marker helps to estimate the risk of cardiovascular diseases in healthy persons, as well as complications in the postinfarction period [42]. Currently, antibodies to cTnI have been developed that demonstrate low cross-reactivity and high specificity [43], and they are commercially available. Despite a considerable number of techniques proposed for cTnI registration [44], the extremely high requirements to detection of this substance still dictate the demand for new, faster and more sensitive approaches. Therefore, the purpose of this research was creating a tool for quantitative real-time monitoring of all stages of immunoassays to accelerate and simplify the development of a wide spectrum of detection techniques.

**Comparative analysis of different schemes of antibody immobilization**

**Fig. 2:**
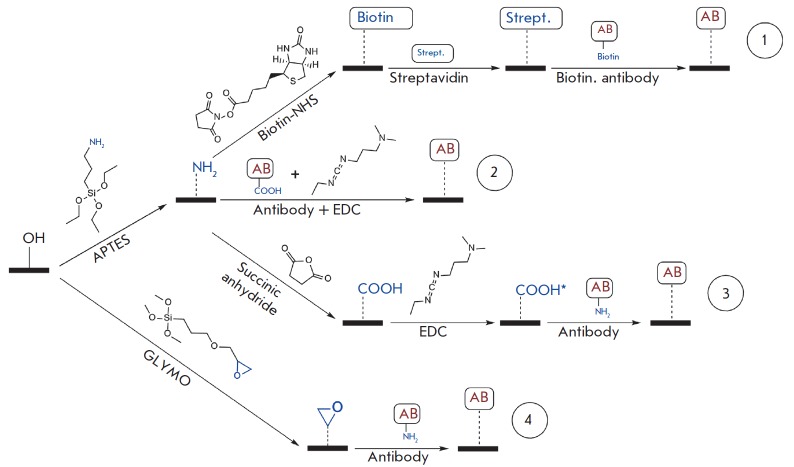
Schemes of antibody immobilization on a glass surface: non-covalent sorption of biotinylated antibody on a biotinylated surface (1); covalent sorption on aminated (2), carboxylated (3) and epoxylated (4) surfaces

The conventional method of sandwich immunoassay comprises several stages: a) antibody immobilization on the surface of a sensor chip (in commercial tests, this is usually done beforehand); b) antigen binding to the immobilized antibody; c) recognition of another epitope of the antigen by tracer antibody; d) association of labels with the tracer antibodies and detection of the labels. The assay characteristics largely depend on the surface chemistry of antibody immobilization on the solid phase. A comparative analysis of four different schemes of glass surface functionalization shown in *Fig. 2* was performed with the use of a Picoscope^®^ biosensor.

**Fig. 3:**
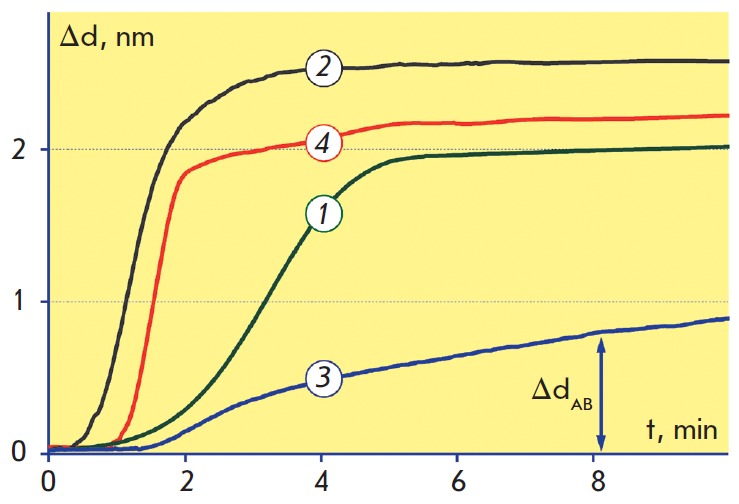
Sensograms of antibody immobilization on biotinylated (1); aminated (2), carboxylated (3) and epoxylated (4) surfaces

The sensograms (dependence of the biolayer thickness on the sensor chip upon time) for the abovemen tioned schemes at the stage of antibody immobilization are shown in *Fig. 3*. These sensograms allow one to estimate the kinetic parameters and integral density of antibody sorption. From this figure we notice that the maximum immobilization rate and the highest sorption density for the selected conditions are achieved with schemes 2 and 4, while scheme 3 features slower sorption and lower immobilization density. These results demonstrate quantitative real-time monitoring of the antibody immobilization process with the proposed approach. Meanwhile, an unambiguous comparison of the selected schemes is beyond the scope of this work as not all the conditions have been preliminarily optimized.

It is worth noticing that the integral density of antibody sorption estimated at the immobilization stage by the change in the biolayer thickness Δd_AB_ may differ from the density of biologically active antibodies on the solid phase surface, because the antibodies may lose their ability to bind antigen during sorption due to partial denaturation, steric inaccessibility of binding sites during the unoriented sorption, etc. As an example, the antibody inactivation may occur while using scheme 2 due to the formation of crossed peptide bonds between different molecules after incubation with carbodiimide. As it is shown below, the SCI method allows one to quantitatively estimate the loss of antibody activity under various immobilization schemes by registration of antigen binding.

It should be noted that schemes 2 and 3 require preliminary activation of the sensor chip surface or antibodies by carbodiimide. Besides time consumption, this activation may lead to insufficient reproducibility of the results in practical conditions when maintaining carbodiimide stability is challenging. Schemes 1 and 4 were chosen for subsequent experiments because of their efficiency and ease of use in laboratory conditions.

**Optimization of protocols for antibody immobilization**

**Fig. 4:**
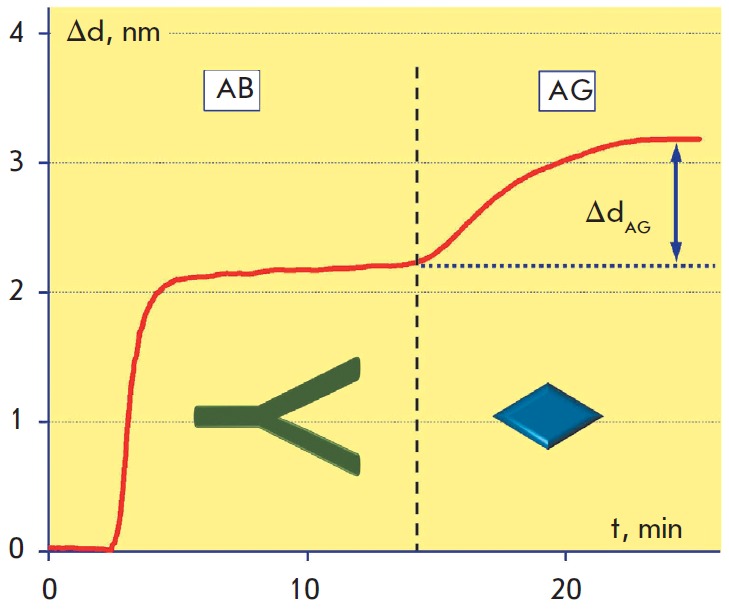
Sensogram of label-free detection of 0.5 μg/ml cardiac troponin I on an epoxylated surface. PBS was passed for 3 min for washing right before the analyte solution (dashed line)

The protocols for antibody immobilization have been optimized to achieve a high sorption capacity of the sensor surface, which is an essential factor for immunoassay sensitivity. The representative sensogram shown in *Fig. 4* illustrates the consecutive changes in the biolayer thickness in response to antibody immobilization on the epoxylated surface and binding of cardiac troponin from the analyte solution containing 0.5 µg/ml cTnI. 3-min PBS washing before inlet of the analyte solution corresponds to a short horizontal fragment of the sensogram, where the biolayer thickness is virtually unchanged.

As a criterion of optimization of the antibody immobilization process, we chose the maximum change in the biolayer thickness Δd_AG_ registered while passing 1 µg/ml antigen solution over the surface of the sensor chips prepared by both methods. The Δd_AG_ value is proportional to the quantity of antigens bound to the antibodies immobilized on the sensor chip surface and characterizes the density of biologically active antibodies on the solid phase surface, which is a more important parameter for the assay sensitivity than the integral density of the immobilized antibodies.

**Fig. 5:**
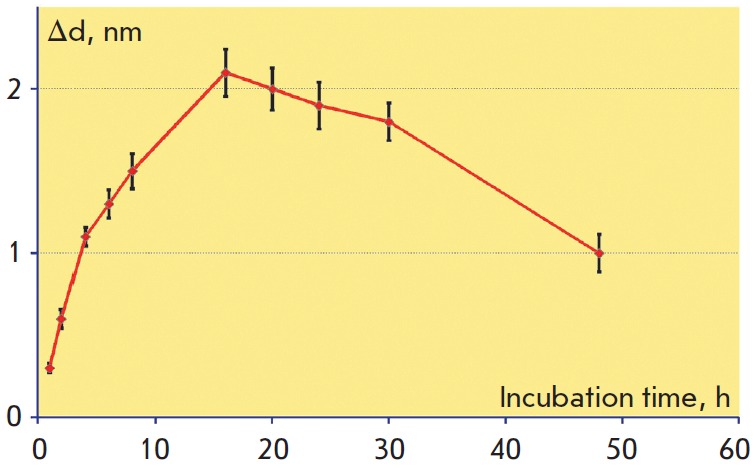
Dependence of the signal recorded during sorption of 1 μg/ml cardiac troponin on the duration of initial epoxylation of the sensor chips in GLYMO at a 0.1% volume concentration of water

For the selected schemes, we studied the dependence of antibody immobilization efficiency on the time of initial incubation of the glass slips with the surfacemodifying agents (APTE S and GLYMO), as well as on the volume concentration of water in the incubation solution. The latter parameter affects the ratio of surface modification rates, copolymerization of organosilanes molecules in the solution, and hydrolysis of their functional groups [45] that define the efficacy of the subsequent immobilization of antibodies: their surface density (optical thickness) and reactivity. It has been shown that the maximum increase in the antigen layer at the stage of immunoassay is achieved if the initial modification of the cover slips lasts 16 h and the volume concentration of water is 1% and 0.1% for APTE S and GLYMO, respectively. To illustrate, the representative plot of the signal versus incubation time for GLYMO is shown in *Fig. 5*. One should note that the maximum biolayer thickness and, thus, the assay sensitivity were achieved if the sensor surface modification was 16 h. The shape of this experimental curve can be explained by the combined behavior of 2 competing processes: immobilization of organosilanes on the sensor chip surface and hydrolysis of the silane epoxy group.

It has been experimentally demonstrated that under optimal parameters the epoxylated and biotinylated surfaces have a virtually identical density of antibody immobilization Δd_AB_, while the density of cTnI antigen sorption on antibody on the epoxylated surface is approximately twice as high; i.e. the proportion of active antibody molecules in that case is substantially higher.

**Calibration curves in label-free mode**

**Fig. 6:**
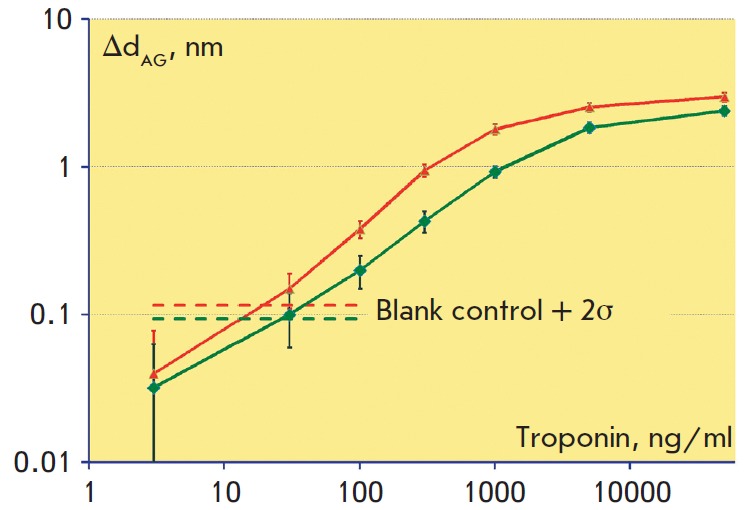
Calibration curves in log-scale obtained in a labelfree mode on biotinylated (*bottom green curve*) and epoxylated (*top red curve*) surfaces show the dependence of the biolayer increase Δd_AG_ upon the cardiac troponin concentration. The horizontal dashed lines represent values that exceed the negative control values by two standard deviations of the negative control in the absence of the antigen

Calibration curves in the label-free mode represent the dependences of the recorded signal Δd_AG_ on the troponin concentration (*Fig. 6*). The detection limits for immobilization on the epoxylated and biotinylated surfaces of the sensor chips were 20 and 30 ng/ml, respectively. The difference in the detection limits for these schemes of antibody immobilization is due to the abovementioned experimental finding that the density of immunoactive antibodies on the epoxylated surface is on average twice higher. The dynamic range in both cases was around 2 orders of concentration magnitude, allowing one to detect troponin levels up to ~3000 ng/ml. This range permits identification of extensive myocardial infarctions but do not cover the entire clinically significant range of low concentrations of cardiac troponin.

The developed protocols of antibody immobilization, as well as the methods for quantitative assesment of immobilization efficiency, can be directly transferred to modern biosensing platforms, including those based on the detection of colored, enzyme, or fluorescent labels. It is noteworthy that Picoscope® allows deposition on the glass slips of a broad range of partially transparent films (polymeric [30], carbonic, and others) or interface layers; i.e. detecting of intermolecular interactions either directly on glass or on many other surface types commonly used in various biosensors. Thus, it is possible to simplify the development of a wide spectrum of different immunoassay types with the use of Picoscope^®^ for the procedures proven to be labor-consuming in label-based methods; e.g., antibody screening, selection of buffer solutions, of incubation times for the reagents, and other stages of preliminary assay optimization. The results of this optimization may then be used in combination with various label-based detection methods. This points to the advantage of the SCI method over the majority of other optical label-free methods and speaks for its usage for the development of a wide range of other biosensing systems.

**Characterization and real-time monitoring of magnetic nanoparticles binding**

**Fig. 7:**
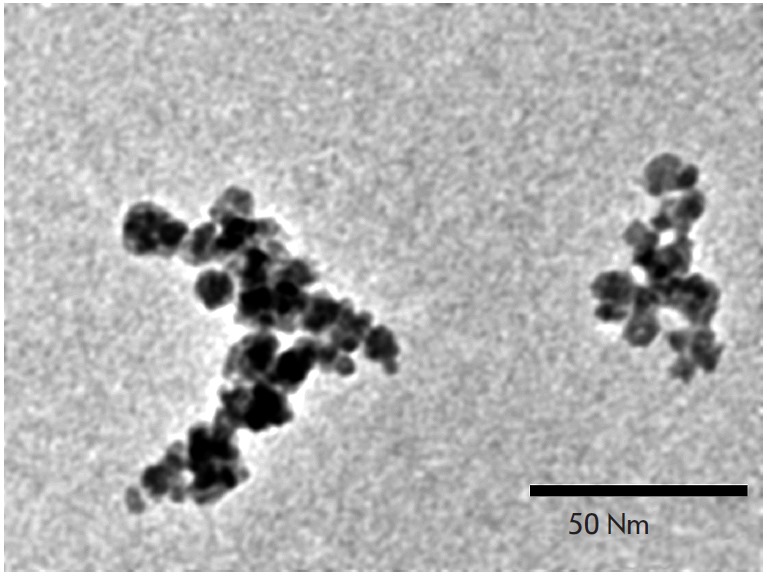
TEM image of streptavidin-conjugated magnetic nanoparticles

The final stage of sandwich immunoassays is the binding of labels to the solid phase and their subsequent detection. In this work, magnetic nanoparticles were used as labels. Application of MNP in immunoassay allows to reduce the assay duration, increasing its sensitivity, enables detection in complex media and analysis of large-volume samples [35]. The representative microphotography of streptavidin-conjugated MNP obtained by a transmission electron microscope is shown in *Fig. 7*. From this figure it can be seen that the particles represent heterogeneous in size clusters consisting of several nanoparticles with a diameter of ~10 nm. This corresponds to the results of several other studies of similar particles [46]. Nevertheless, these particles show low nonspecific binding to the components of complex biological mediums (e.g., whole blood [47]) and ensure high reproducibility of immunoassay results when used as the detection labels [35].

**Fig. 8:**
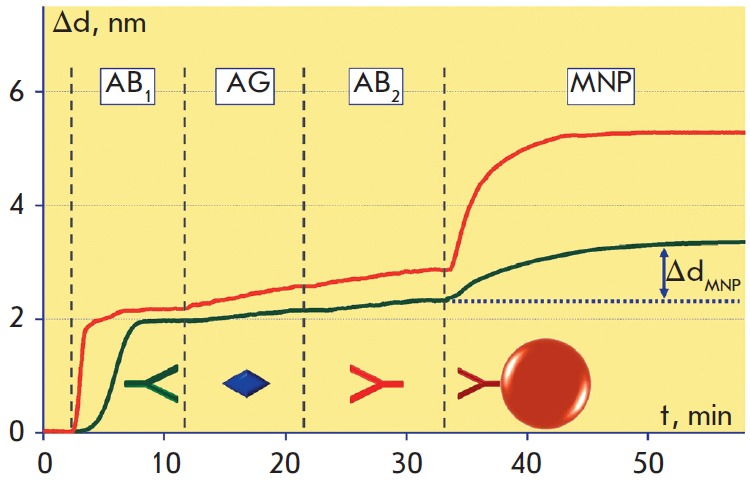
Sensograms demonstrating all stages of the magnetic immunoassay on the biotinylated (*bottom green curve*) and epoxylated (*top red curve*) surfaces of the sensor chip: AB_1_ – antibody immobilization; AG – antigen (100 ng/ml) capture by immobilized antibodies; AB_2_ – recognition by tracer antibodies of another antigen epitope; MNP – association of magnetic nanoparticles with tracer antibodies. PBS washing was performed before each step as indicated with dotted lines

*Figure 8 *shows the sensograms obtained for chips with epoxylated (upper curve) and biotinylated (lower curve) surfaces that demonstrate all stages of a magnetic sandwich immunoassay with detection of a 100 ng/ml cTnI solution. Washing by PBS implemented right before the injection of each immunoreagent corresponds to the short horizontal sections of the sensograms when the biolayer thickness remains practically unchanged. In the assay on the epoxylated chips, covalently immobilized native capture antibodies AB1, biotinylated tracer antibodies AB2, and MNP covered with streptavidin were used. The use of such magnetic particles together with the biotinylated sensor chips might cause high nonspecific signals due to direct binding of MNP with free biotin molecules. Therefore, AB1 antibodies of the IgG2b isotype were immobilized on the biotinylated chips via streptavidin, and AB_2_ of the IgG1 isotype were used as tracer antibodies, along with another type of magnetic particles that specifically recognize this AB_2_ isotype.

The differences at the AG and AB2 stages in the sensograms obtained for different surfaces with the same antigen concentration become more prominent at the MNP stage. *Figure 8 *illustrates that the binding rate of streptavidin-conjugated MNP is higher than that of MNP covered with antibodies to the isotype of the tracer antibody. First, this can be explained by the different efficiency of antibody sorption on the sensor chip. Second, the kinetic constant of association for biotin- streptavidin is several orders of magnitude higher than that of the monoclonal antibody with antigen. This provides stronger and faster binding of MNP covered with streptavidin, compared with MNP covered with antibodies.

The obtained results allow estimation of the kinetic characteristics of interactions between biomolecules, as well as with magnetic particles. The observed kinetic association constants estimated for each assay stage by the sensograms recorded by Picoscope^®^ are shown in the table. These values observed at the MNP stage are 2–3 orders of magnitude higher than those at the preceding stages for both schemes of antibody immobilization, despite the fact that at each subsequent assay stage, this parameter is smaller than its true value due to dissociation of the complexes formed at the previous stage. Such good kinetic characteristics of MNP binding compared to antibody and antigen molecules may be explained by MNP polyvalence. Several biorecognition biomolecules simultaneously linked to a single particle may provide a higher probability of effective collision of MNP with the sensor chip surface.

**Fig. 9:**
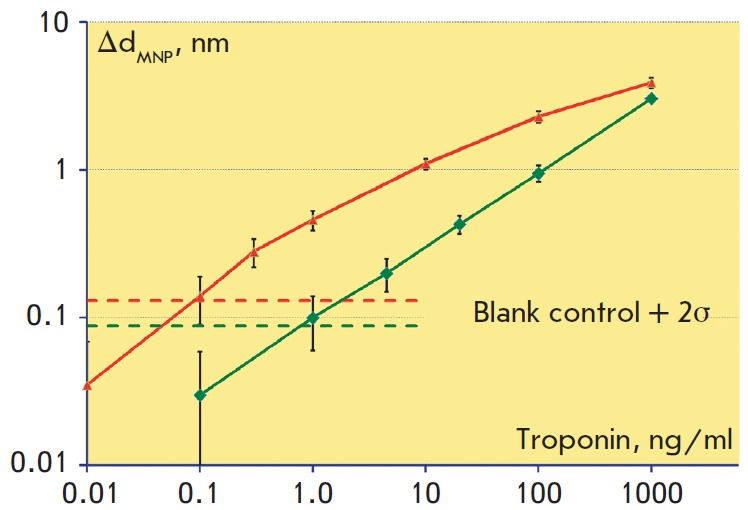
Calibration curves in log-scale obtained while detection of cardiac troponin at the stage of magnetic nanoparticles passing along the biotinylated (*bottom green curve*) and epoxylated (*top red curve*) surfaces of the sensor chip. The horizontal dashed lines represent values that exceed the negative control values by two standard deviations of the negative control in the absence of the antigen

*Figure 9 *shows the dependences of the biolayer increase Δd_MNP_ upon troponin concentration at the stage of MNP passing. The detection limit on the epoxylated surface calculated using the 2σ criterion was 0.1 ng/ml, which was 10 times better than the 1 ng/ml value obtained for the biotinylated surface. The dynamic range in both cases was around 3 orders of concentration magnitude. Thus, the use of MNP provides a resulting improvement of the detection limit of over 100 times as compared with label-free detection, i.e. multiple amplification of the signal is realized. The significantly greater increase in the biolayer thickness Δd_MNP_ at the MNP stage compared to the stages of antigen and detecting antibody binding over the full range of measured cTnI concentrations is due to the fact that the nanoparticle diameter is significantly larger than the characteristic sizes of the detected biomolecules. The achieved detection limit for cardiac troponin of 0.1 ng/ml corresponds to the clinically significant threshold for the diagnostics of myocardial infarction [48]. The high sensitivity and wide dynamic range make the developed biosensor an attractive instrument with affordable consumables (disposable sensor chips) for real-time immunoassays in disease diagnostics, detection of pathogens in food, and environmental monitoring. Further studies will aim at estimating the efficiency of cTnI detection in real biological samples and validating the correlation between the obtained results and the data received by conventional methods.

**Table:** Dependence of the kinetic association constant observed at each stage of the magnetic immunoassay upon the surface type

Surface type	Observed kinetic association constant, M^-1^s^-1^		
	AG stage	AB_2_ stage	MNP stage
Epoxylated	(6.4 ± 1.3) × 10^5^	(1.2 ± 0.2) × 10^5^	(1.6 ± 0.2) × 10^8^
Biotinylated	(8.2 ± 1.9) × 10^5^	(1.7 ± 0.3) × 10^5^	(6.4 ± 1.1) × 10^7^

Beside the dignostic significance of the developed immunoassay achieved due to the amplification of the SCI signal, our results may be of special interest for the investigation and kinetic characterization of the interactions of nanoparticles with molecules. We should note that to date the most widespread label-free biosensors that allow estimation of the kinetic parameters of nanoparticles are those based on the surface plasmon resonance (SPR) [21, 15]. These biosensors permit the study of interactions of particles with the biomolecules immobilized on the modified surface of highly conductive gold or silver films. The interface (surface) chemistry used in this case significantly differs from that used in the most widespread methods of solid-phase immunoassay. This fact complicates the transfer of the results obtained with the SPR biosensors to other platforms. As a contrast, the SCI method in combination with inexpensive, disposable sensor chips greatly enhances research opportunities for the development of various immunoassays. The methods for immunoassay optimization proposed in this work allow easy transfer of all the protocols to label-based biosensing platforms; for example, those using the highly sensitive methods of MNP detection by compact electronic devices [15], based on frequency mixing [49] under nonlinear particle remagnetization [34].

The dependence of the signal amplification efficacy on the MNP size deserves a separate study. On the one hand, bigger magnetic particles (up to several micrometers) can better amplify the signal. However, bigger particles are prone to gravity sedimentation and may cause a high nonspecific signal. Neither sedimentation nor nonspecific interaction with the surface was observed in this work while using 50 nm MNP; therefore, the use of smaller nanoparticles appears to be unreasonable.

The absence of nonspecific sorption during signal amplification permits further gain in sensitivity by using several amplification steps. As an example, if a biotinylated protein with several biotinylation sites is repeatedly passed between two runs of streptavidin- conjugated MNP, it is possible to substantially increase the number of labels and, hence, the sensitivity.

In addition to low nonspecific binding, MNP possess unique properties that can be used to further develop the proposed techniques. For example, a combination of magnetic properties with optical detection allows one to realize an optomagnetic immunoassay [50]. In that assay, the duration of immunochemical reactions can be significantly reduced due to magnetic stirring by MNP, as well as by rotation of MNP chains by a magnetic field. It also permits one to enrich the sample with antigens by magnetic separation and to decrease nonspecific binding of labels with the surface by removal of the loosely bound particles by a magnetic field of the respective spatial orientation for “magnetic” washing of the labels.

## CONCLUSION

We have developed a method for magnetic immunoassay on a glass surface that allows real-time detection of each stage of the assay and usage as disposable sensor chips of inexpensive cover slips without deposition of any films . Four schemes of antibody immobilization have been tested and optimized with their efficacy assessed. As a result, high sorption capacity and proportion of active antibodies on the surface of the sensor chip have been achieved. The possibility of real-time recording of the kinetics of interactions of magnetic nanoparticles with biomolecules has been shown. It has been demonstrated that the use of magnetic nanoparticles amplifies the signal of spectral correlation interferometry, which leads to an additional 100-fold improvement of the detection limit for cardiac troponin. Thus, the developed interferometric biosensing systems may serve as effective tools for conducting the assays, as well as for a wide range of applications, including the development and optimization of immunoassay methods, quality control of immunoreagents, and control of surface sorption properties. The obtained results on the selection of schemes and optimization of antibody immobilization protocols, as well as on realtime monitoring of all immunoassay stages and kinetic characterization of nanoparticles, can be directly transferred to other biosensing platforms, including those based on various labels – magnetic, fluorescent, enzymatic, and others. The proposed biosensing technique is an economically sound alternative for immunoassays with disposable consumables for disease diagnostics, detection of pathogens in food, and environmental monitoring.

## Abbreviations

AR: analytical grade
APTES: (3-Aminopropyl)triethoxysilane
BSA: bovine serum albumin
cTnI: cardiac troponin I
DMF: dimethylformamide
GLYMO: (3-glycidyloxypropyl)trimethoxysila
MNP: magnetic nanoparticles
PBS: phosphate buffered saline
SCI: spectral correlation interferometry
